# Rapid-response, low-detection-limit, positive-negative air pressure sensing: GaN chips integrated with hydrophobic PDMS films

**DOI:** 10.1038/s41378-024-00766-6

**Published:** 2024-11-01

**Authors:** Sizhe Gui, Binlu Yu, Yumeng Luo, Liang Chen, Kwai Hei Li

**Affiliations:** 1 School of Microelectronics, Southern University of Science and Technology, Shenzhen, 518055 China; 2 Foshan Electrical and Lighting Company Ltd., Foshan, 528000 China

**Keywords:** Optical sensors, Electrical and electronic engineering

## Abstract

Despite the importance of positive and negative pressure sensing in numerous domains, the availability of a single sensing unit adept at handling this dual task remains highly limited. This study introduces a compact optical device capable of swiftly and precisely detecting positive and negative pressures ranging from −35 kPa to 35 kPa. The GaN chip, which serves as a core component of the device, is monolithically integrated with light-emitting and light-detecting elements. By combining a deformable PDMS film coated with a hydrophobic layer, the chip can respond to changes in optical reflectance induced by pressure fluctuations. The integrated sensing device has low detection limits of 4.3 Pa and −7.8 Pa and fast response times of 0.14 s and 0.22 s for positive and negative pressure variations, respectively. The device also demonstrates adaptability in capturing distinct human breathing patterns. The proposed device, characterized by its compactness, responsiveness, and ease of operation, holds promise for a variety of pressure-sensing applications.

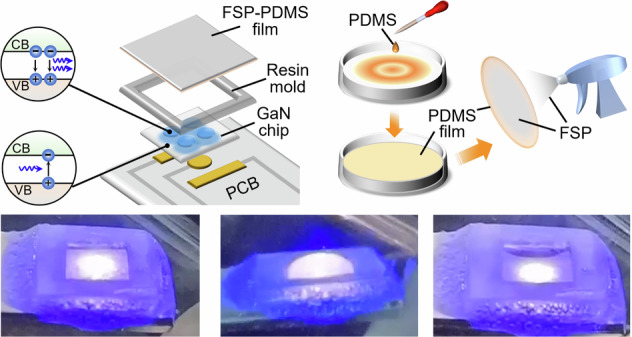

## Introduction

Air pressure sensing holds immense significance across various applications, ranging from altitude sensing and fluidic flow detection to leakage inspection and health care monitoring^1–6^. Notably, there is a growing demand for air pressure sensors capable of attaining a low detection limit below 10 Pa for both positive and negative pressures, particularly in health care and medical applications^7,8^. To meet this need, various technologies have been extensively researched, including those based on piezoelectric^9,10^, piezoresistive^11,12^, triboelectric^13,14^, and capacitive^15,16^ principles. Despite the progress made in nanostructures and microfabrication techniques to enhance sensing capabilities, the challenge persists in developing a compact, swiftly responsive sensor with a low detection limit for both positive and negative pressures.

Optical approaches provide promising alternatives to electrical sensing methods because of their inherently rapid response and high sensitivity^17–20^. However, current optical sensing techniques often depend on bulky optical components, impeding their miniaturization^21–23^. Recently, electrical‒optical‒mechanical systems have attracted considerable attention owing to their fast switching ability, high sensitivity, small footprint, and potential adaptability in harsh environments. For example, microelectromechanical system (MEMS) fiber‒optic pressure sensors that employ Fabry‒Pérot (FP) interferometry are capable of detecting pressures ranging from hundreds of kPa to MPa^24–26^. However, their operation focuses mainly on positive high pressure, but they are not sensitive to small changes in positive and negative pressures. Furthermore, the intricate integration process of the FP cavity with optical fibers often necessitates specialized packaging components and precise optical alignment, further increasing the overall complexity.

To overcome this limitation, on-chip photonic integration has emerged as a viable solution. Among various material platforms, GaN semiconductors and their alloys stand out because of their exceptional properties, such as high efficiency, prolonged lifespan, and high stability^27–29^. Despite the commercial availability of various GaN-based light-emitting diodes (LEDs) and photodetectors (PDs), their compact integration into functional sensing systems remains challenging because of the incompatibility of their device structure and manufacturing process, necessitating additional off-chip assembly procedures. Recently, on-chip integration of GaN-based optical devices has been extensively researched for visible light communication^30–32^, but the development of a sensing unit capable of positive-negative air pressure sensing remains unexplored.

In this work, a compact optical pressure-sensing device is proposed through the compact integration of a GaN chip and a polydimethylsiloxane (PDMS) film. The GaN chip, which incorporates LEDs and PDs onto a single chip-scale platform, is fabricated via wafer-scale manufacturing processes. An ultrathin PDMS film is created via a straightforward and cost-effective floating-on-water method, which requires simpler equipment as compared with conventional spin-coating techniques^33–35^. This method can eliminate the step of peeling from a substrate, and the PDMS film can be readily transferred from the water surface to another substrate while maintaining its integrity. Compared with other materials, such as polyethylene terephthalate and polyimide, polydimethylsiloxane (PDMS) has a smaller Young’s modulus, making it a more suitable candidate for developing thin films that undergo significant deformation in response to small pressure variations. However, the surface viscosity of ultrathin PDMS films becomes more pronounced, potentially posing significant hysteresis challenges^36–38^. To circumvent this issue, a hydrophobic coating is introduced onto the PDMS film, which not only mitigates hysteresis by rendering the film surface hydrophilic but also enhances its optical reflectance. Moreover, to facilitate compact integration of the PDMS film, a flip‒chip bonding technique is employed instead of conventional wire bonding for packaging of the GaN chip. The properties of the integrated device are thoroughly investigated, demonstrating the effectiveness of the miniaturization design.

## Results and discussion

### Integration of the GaN chips with hydrophobic PDMS films

**Fig. 1:**
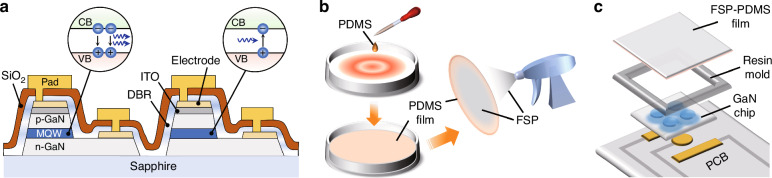
Structure of the sensing device. **a** Schematic diagram of the GaN device from a cross-sectional view. **b** Schematic diagram of the fabrication process of the FSP-PDMS film. **c** Schematic diagram of the sensing device

Figure 1a shows a schematic diagram of a GaN chip consisting of an LED and a PD with identical device structures. Figure 1b depicts the fabrication process of a PDMS film via the floating-on-water method. After applying the fluorosilane polymer (FSP) coating on the PDMS film, the FSP-PDMS film is affixed to a resin mold created via a 3D printer. This mold is used to shape the suspended region of the film and assemble the GaN chip bonded on the PCB, as depicted in Fig. 1c.

**Fig. 2:**
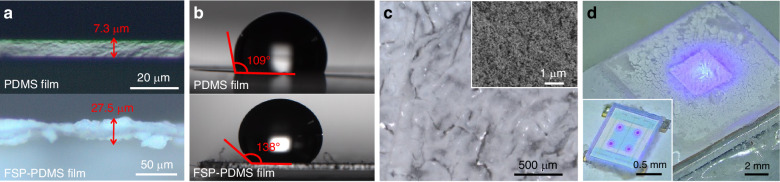
Morphology of the sensing device. **a** Cross-sectional micrographs of the PDMS and FSP-PDMS films. **b** Optical images showing the contact angles of the PDMS and FSP-PDMS films. **c** Optical image of the FSP-PDMS film captured by confocal microscopy. The inset shows an image captured via SEM. **d** Optical image of the resulting sensing device. The inset shows an optical image of the GaN chip

The thickness of the FSP-PDMS film was measured via an optical microscope (Olympus BX53M). As shown in the cross-sectional image in Fig. 2a, the PDMS film has a thickness of 7.3 μm prior to FSP coating, which subsequently increases to 27.5 μm after the coating is applied. Figure 2b shows the water drop contact angle measurements conducted on the PDMS film, comparing its behavior with and without the FSP coating. After the FSP coating was applied, the contact angle of the film increased to 138°, indicating an increase in its hydrophobicity. Figure 2c shows the surface morphology of the film examined via a confocal microscope (VK-X 5000), which yielded a value of 1.62 μm for surface roughness. In addition, the inset of Fig. 2c, which was captured by scanning electron microscopy (SEM) (Zeiss Supra 55, Germany), reveals that the particle size is ~100 nm. The inset of Fig. 2d illustrates the configuration of the GaN device, highlighting the four 0.2-mm-diameter circles that function as the LED, whereas the surrounding chip area with a size of 1.1 × 1.3 mm^2^ serves as the PD. Figure 2d shows the resulting sensing sensor after the GaN chip was integrated with the FSP-PDMS film.

### Operational principle of the sensing device

**Fig. 3:**
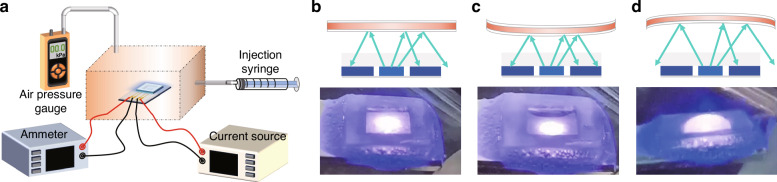
Working principle of the sensing device. **a** Schematic diagram of the measurement step. Schematic diagrams and optical images of the sensing device under **b** normal pressure, **c** positive pressure, and **d** negative pressure

The experimental measurement setup is illustrated in Fig. 3a. A custom-made polypropylene chamber was prepared. The air pressure inside the chamber is adjusted via an injection syringe, and the pressure in the chamber is calibrated via a commercial digital barometer (Benetech GM520). All the connections to the chamber are sealed with vacuum sealant to ensure the airtightness of the entire system. During the measurement process, the LED and PD of the GaN chip are connected to a current source (Keithley 2450) and an ammeter (Keithley 6500), respectively.

Figure 3b–d schematically depicts the operational principle of the sensing device. Under forward bias conditions of the LED, the recombination of electrons and holes within the InGaN/GaN multiquantum wells (MQWs) gives rise to light emission. The light emitted from the LED transmits through the transparent sapphire substrate and reaches the FSP-PDMS film. The FSP coating partially reflects the incident light, directing it toward the PD, where it is absorbed, generating a photocurrent, as shown in Fig. 3b. As the ambient pressure increases, the FSP-PDMS film approaches the GaN chip, increasing the proportion of light reaching the PD, as depicted in Fig. 3c. Conversely, a decrease in ambient pressure deforms the film upward, as illustrated in Fig. 3d, leading to a reduction in the amount of light captured by the PD. This change in light intensity modulates the photocurrent produced by the PD, providing a quantifiable measure of pressure variations.

### Characterization of the GaN chip

**Fig. 4:**
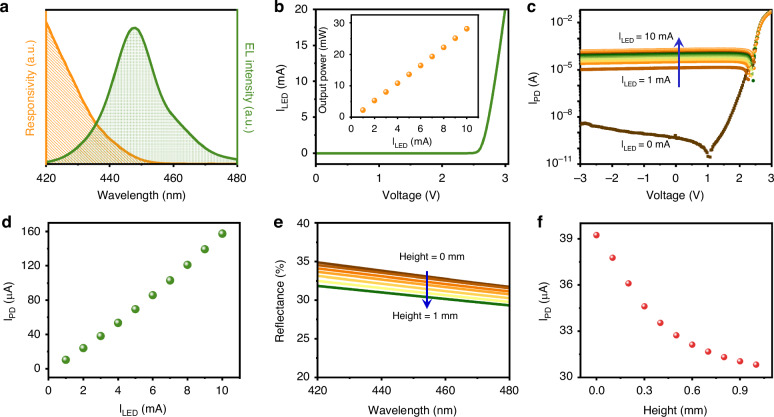
Properties of the GaN chip. **a** Plot of the absorption spectrum of the PD and emission spectrum of the LED. **b** I–V characteristics of the LED. The inset shows the output power of the LED as a function of the driving current. **c** I‒V curves of the PD measured at different LED driving currents. **d** Plot of the photocurrent of the PD as a function of the driving current of the LED. **e** Reflectance spectra of the FSP-PDMS film after different separations. **f** Plot of the photocurrent as a function of the height of the FSP-PDMS film

Figure 4 presents an analysis of the electrical and optical properties of the GaN chip, which were measured via a source meter (Keithley 2450) with a resolution of 50 pA. Figure 4a shows the emission spectrum of the LED, operating under a 2-mA driving current, alongside the absorption spectrum of the PD. The LED has a peak wavelength of 448 nm, accompanied by a full width at half-maximum (FWHM) of 16 nm. Notably, the absorption curve overlaps with the LED emission spectrum within the spectral range of 420–450 nm^39^. Figure 4b shows the current‒voltage (I‒V) curve of the LED, revealing a forward-biased voltage of 2.34 V when driven by a current of 2 mA. The power consumption is determined to be 4.68 mW. The inset of Fig. 4b further shows the direct proportional relationship between the light output power of the LED and the driving current. The GaN chip also exhibited a fast transient response, with a rise time of 5.0 μs and a fall time of 4.7 μs (see Supporting Information S1). Notably, the LED-PD chip incorporating InGaN/GaN MQWs, operating at a shorter wavelength, exhibits a greater extent of overlap between its emission and absorption spectra^40^, thereby potentially enhancing its light detection capabilities. However, given the safety considerations related to prolonged UV exposure, the chip is intentionally designed to operate within the blue visible spectral range.

The I‒V curves of the PD recorded under different LED driving conditions are plotted in Fig. 4c to verify the light-detecting characteristic of the PD. Under dark conditions, the photocurrent measured at a reverse bias voltage remains low at ~10^−9 ^A. When the LED is driven with a 2 mA current, the photocurrent increases prominently to ~10^−5 ^A, indicating a strong responsivity of the PD to the LED emission. The noise magnitude is on the order of 10^−9^–10^−10^ A (see Supporting Information S2). Figure 4d presents a plot of the photocurrent versus the LED driving current, clearly demonstrating the linear response of the PD. This linearity ensures reliable and accurate detection of variations in light intensity.

### Optimization of the FSP-PDMS film

Given that the bare PDMS film is optically transparent and viscous, it is inadequate as an efficient light reflector for responding to pressure variations. The introduction of an FSP layer serves to overcome these limitations; however, the thickness of this layer is a pivotal factor in determining both its morphology and optical reflectance (see Supporting Information S3 and S4). With a coating thinner than 5.8 μm, the film fails to form a contiguous layer over the entire surface, allowing light to escape through uncovered regions, thereby diminishing the reflected light intensity. Conversely, when a thicker FSP coating exceeding 14.8 μm is applied, undesirable agglomeration and cracking occur within the coating, leading to light leakage. Therefore, to ensure seamless coverage on the PDMS without discernible cracking, the thickness of the FSP layer was optimized to a moderate value of 10.1 μm.

As the proximity of the film plays an important role in converting the pressure changes into optical variations, its reflectance properties at varying positions are measured. The distance between the film and the fiber port is precisely controlled via a linear motorized stage, whereas the input fiber port is connected to a broadband light source. Subsequently, the reflectance signal is fiber coupled to a spectrometer. Unlike the bare transparent PDMS film, which exhibited negligible reflectance (see Supporting Information S5), the introduction of FSP layers onto the PDMS film resulted in a substantial increase in reflectance, exceeding 30%. Figure 4e shows the measured reflectance of the FSP-PDMS film at various positions above the GaN chip, revealing that the amount of received light decreases from 32.7% to 30.2% as the distance decreases from 1 mm to 0 mm. This observation implies that a significant portion of the incident light is being reflected, further confirming the effectiveness of the FSP layers. To further verify this, Fig. 4f presents the photocurrents of the PD measured at different heights of the FSP-PDMS film above the GaN chip. As the height decreases from 1 mm to 0 mm, the photocurrent increases from 30.82 to 39.24 μA.

### Response of the sensing devices to pressure

The response of the sensing device to both positive and negative pressures is characterized. The device is placed in a closed chamber that is equipped with two ports. One port is interfaced with an air gauge to introduce positive pressure, whereas the other port is linked to an air compressor to generate negative pressure. During the measurement, the LED on the GaN chip is supplied with a driving current of 2 mA through a source meter (Keithley 2450). Simultaneously, an ammeter (Keithley 6500) is connected to the PD to record the photocurrent signal.

**Fig. 5:**
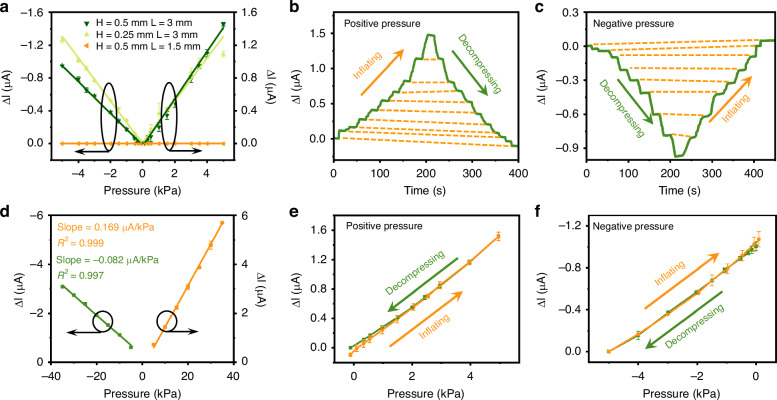
Response of the sensing device to positive and negative pressure. **a** Plot of the photocurrent variation as a function of pressure for FSP-PDMS films of different sizes. Measured photocurrent changes under stepwise changes in **b** positive and **c** negative pressure. **d** Plot of the photocurrent variation as a function of pressure from −35 kPa to 35 kPa. Photocurrent variation versus pressure curve under **e** positive and **f** negative pressure

Given that the chip can respond strongly to films positioned within the submillimeter range above the chip, the initial position and size of the suspended area of the film are important factors affecting the sensing performance. Experimentally, the films with varying combinations of height (H) and side length (L) are defined by the resin molds (see Supporting Information S6). Figure 5a shows the response of the devices across a pressure range of −5 kPa to 5 kPa when varying H and L are applied. The PDMS film has a high Young’s modulus^41–43^. Together with the FSP layer, the small suspended film with L = 1.5 mm has a limited deformation ability, thus restricting the ability of the chip to respond to changes in air pressure. By increasing the suspended area to L = 3 mm, the response is significantly enhanced, reaching 1.46 μA at 5 kPa and −0.95 μA at −5 kPa. Although reducing H from 0.5 mm to 0.25 mm results in an enhanced response due to the proximity of the film to the chip surface, the increase in the photocurrent saturates at pressures exceeding 4 kPa.

To establish a balance between the sensitivity and measurement range, the optimal device configuration is H = 0.5 mm and L = 3 mm. This configuration demonstrates favorable performance characteristics, with a slope of 0.285 μA/kPa and R^2^ of 0.997 under positive pressures from 0 kPa to 5 kPa, along with a slope of −0.195 μA/kPa and R^2^ of 0.999 under negative pressures from −5 kPa to 0 kPa. Figure 5d shows the photocurrent response under extended pressures up to −35 kPa and 35 kPa, revealing sensitivities of −0.082 μA/kPa and 0.169 μA/kPa for negative and positive pressures, respectively.

The photocurrent responses of the device to dynamic pressure variations are investigated. Figures 5b and 5c depict the photocurrent response of the device when subjected to stepwise increases and decreases in both positive and negative pressures, ranging from 0 to 5 kPa and 0 to −5 kPa, respectively. The profiles presented here exhibit a high degree of symmetry, indicating a sensitive and consistent response from the device. Moreover, an analysis of the inflation and decompression processes depicted in Fig. 5e, f reveals hysteresis values of 3.64% and 6.13% for positive and negative pressures, respectively. This low hysteresis is attributed primarily to the presence of the FSP coating on the film, which effectively decreases the surface viscosity of the PDMS.

### Dynamic response of the sensing device

**Fig. 6:**
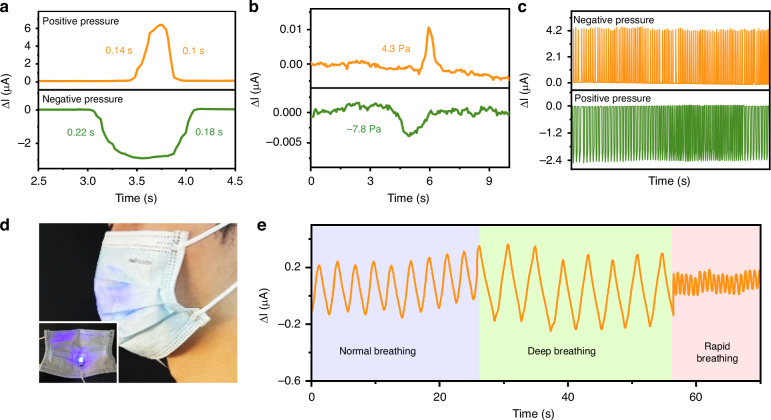
Dynamic response and application of the sensing device. **a** Transient photocurrent response showing the response and recovery time of the device. **b** Photocurrent response of the device when applying pressures of −7.8 Pa and 4.3 Pa. **c** Reliability measurement of the device. **d** Optical images of the sensing device in a mask. **e** Photocurrent response of the device to different breathing conditions

The transient characteristics of the sensing device are analyzed by subjecting it to instant inflation and deflation. From the transient characteristics of the device shown in Fig. 6a, the response and recovery times under negative pressure are found to be 0.22 s and 0.18 s, respectively, whereas under positive pressure, these values are 0.14 s and 0.1 s, respectively. Figure 6b shows the detection limit of the device, indicating its sensitivity to mild pressures of −7.8 Pa and 4.3 Pa. To evaluate its repeatability, the sensing device undergoes over 100 cycles at both positive and negative pressures at frequencies of 0.19 Hz and 0.54 Hz, respectively. As shown in Fig. 6c, the photocurrent consistently reaches the same peak variation in each cycle before returning to its initial value. Notably, the sensing device is capable of functioning effectively at low frequencies, which are significantly lower than the resonant frequency of the PDMS film, as determined by simulation (see Supporting Information S7).

### Performance analysis of the sensing device

Table 1 presents a comparative analysis between the developed device and previously reported air pressure sensors. While some reported sensors demonstrate low detection limits on the Pa scale and subsecond response times, their operation is confined to the positive pressure domain. Only some studies have managed to achieve detection capability for both the negative and positive ranges; however, simultaneously achieving a wide measurement range, fast response, and rapid response time remains a challenge. On the other hand, the device introduced in this work exhibited superior performance across these parameters. Characterized by low detection limits and reduced response times, the device further boasts an extensive operational range spanning from −35 kPa to 35 kPa. Notably, unlike conventional optical systems, the developed device adopts a chip-scale integration design and utilizes wafer-level fabrication processes. This approach eliminates the need for bulky external optical components, achieving high compactness and substantially reducing the footprint to the millimeter scale.

**Table 1:** Comparison with other reported air pressure sensors

Sensing method	Detection range	Detection limit	Response time	Sensitivity	Size
Triboelectric^1^	−4–17 kPa	2.3 kPa/−1.2 kPa	0.2 s	0.44 V/kPa	3.7 × 3.7 cm^2^
Triboelectric^2^	1–4 kPa	1 kPa	0.28 s	0.46 V/kPa	Tens of mm
Piezoelectric^11^	0–180 Pa	15 Pa	0.15 s	0.19 V/Pa	2.5 × 2.5 cm^2^
Piezoresistance^14^	0–40 kPa	12 Pa	~10 s	0.13 kPa^−1^	–
Piezoresistance^15^	0–30 kPa	1 Pa	0.2 s	9.15 kPa^−1^	15 × 15 mm^2^
Capacitance^18^	−60–20 kPa	2 kPa	4 s	0.15–0.77%/kPa	–
Capacitance^19^	−3–3 kPa	50 Pa/−50 Pa	0.308 s	0.28 kPa^−1^	–
Optical fiber^20^	20–200 kPa	20 kPa	–	303.65 pm/kPa	–
This work	−35–35 kPa	4.3 Pa/−7.8 Pa	0.22 s/0.14 s	0.285 μA/kPa −0.195 μA/kPa	3 × 3 mm^2^

Owing to the low elastic modulus and small thickness of the PDMS film, the suspended area undergoes irreversible distortion and is unable to revert to its initial flat shape upon exposure to pressure exceeding ±60 kPa, leading to mechanical failure. Although increasing the thickness of the PDMS film can be a potential solution for enhancing its resilience to high pressures, this approach induces a tradeoff in sacrificing the sensitivity of the sensing device.

### Application of the sensing device in respiratory monitoring

Owing to its low detection limit and swift responsiveness to both positive and negative pressure fluctuations, the sensing device is well-suited for real-time monitoring of pressure variations. Among the various pressure-sensing applications, monitoring human breathing is considered a particularly demanding task, requiring sensing units with high sensitivity and rapid response across the positive-to-negative pressure range. Considering that human breathing can induce an air pressure change from hundreds of Pa to tens of kPa^44^, the potential applicability of the device in respiratory monitoring is explored. As shown in Fig. 6d, the device is affixed to the mask, enabling the collection of breathing signals. Figure 6e shows a periodic photocurrent signal characterized by distinct periods and amplitudes, confirming its ability to capture different breathing patterns. The frequencies of the measured patterns for normal, deep, and rapid breathing are 0.35 Hz, 0.25 Hz, and 1.43 Hz, respectively. During normal breathing, the photocurrent variation is ~0.3 μA. Under deep breathing conditions, more considerable air pressure changes increase the variation, reaching 0.6 μA. In the case of rapid breathing, the photocurrent variation diminishes and becomes more frequent, reflecting the elevated respiration rate.

## Conclusion

In conclusion, this study demonstrates an air pressure-sensing device that integrates a GaN chip with an FSP-PDMS film. The optimized integration of the FSP-PDMS film endows the device with the ability to respond to an extensive air pressure range spanning from −35 kPa to 35 kPa. It has low detection limits of 4.3 Pa and −7.8 Pa for positive and negative pressures, respectively, while exhibiting swift response times of 0.22 s and 0.14 s. The compact design, rapid response capabilities, and high stability of the device render it a suitable candidate for a variety of pressure-sensing applications across different fields.

## Materials and methods

### Fabrication of the GaN chip

Initially, unintentionally doped GaN, n-GaN, InGaN/GaN MQWs, and p-GaN are epitaxially grown on a 4-inch sapphire substrate via metal‒organic chemical vapor deposition. A 0.12-μm-thick indium tin oxide (ITO) layer is deposited onto the p-GaN layer. A square mesa of 1.1 × 1.3 mm^2^ and four circular mesas, each with a diameter of 0.2 μm, are defined as a PD and an LED, respectively, by photolithography. The unmasked GaN areas are etched to expose n-GaN via inductively coupled plasma (ICP) etching. A subsequent ICP process ensures electrical isolation of the LED and LD by removing the intervening GaN layers. Subsequently, the electrodes are deposited through electron beam evaporation. A SiO_2_ passivation layer is coated, followed by a distributed Bragg reflector (DBR) layer deposited using an optical film coater. Metal pads are coated by electron beam deposition, completing the electrical contacts of the chip. Finally, the sapphire substrate undergoes a polishing and thinning process, and the wafer is diced by laser micromachining.

### Preparation of FSP-PDMS films

A PDMS gel is prepared by mixing an elastomer base with a curing agent in a 10:1 ratio. The mixture undergoes vacuum degassing to eliminate trapped air bubbles. Subsequently, 20 μg of the PDMS gel are gently dispensed onto the surface of the water contained in a Petri dish. Owing to its lower density and inherent immiscibility with water, the PDMS gel floats and spreads evenly across the water surface. The PDMS gel rapidly self-expands over the water surface within 30 s. After allowing the sample to rest for 24 h at room temperature, it transforms into a thin film that floats on the water. The PDMS film is carefully retrieved, air-dried, and subsequently sprayed on both sides with FSP solution at a concentration of 0.8 g/ml. A hot air blower is utilized to accelerate the drying process. The thickness of the PDMS film can be controlled by adjusting the amount of PDMS gel. A discussion of the calibration parameters can be found in Supporting Information S8.

## Supplementary information

Supplementary Information

## Data Availability

The data supporting plots within this paper and other findings of this study are available from the corresponding author upon request.
