# Supplementary material for: Rapid-response, low-detection-limit, positive-negative air pressure sensing: GaN chips integrated with hydrophobic PDMS films
Source: Microsyst Nanoeng. 2024 Nov 1;10:162. doi: 10.1038/s41378-024-00766-6 (PMC11527884; doi:10.1038/s41378-024-00766-6)
Supplement: Supplementary file 1 — Supplementary Information [file 41378_2024_766_MOESM1_ESM.docx]

**Supplementary information**

**Rapid-Response, Low-Detection-Limit, Positive-Negative Air Pressure Sensing: GaN Chips Integrated with Hydrophobic PDMS Films**

**Sizhe Gui^1,#^, Binlu Yu^1,#^, Yumeng Luo^1^, Liang Chen^2^, and Kwai Hei Li^1,*^**

*^1^School of Microelectronics, Southern University of Science and Technology, Shenzhen 518055, China*

*^2^Foshan Electrical and Lighting Company Ltd., Foshan 528000, China.*

*^#^The authors contribute equally*

Email addresses: Sizhe Gui (12132442@mail.sustech.edu.cn), Binlu Yu (12031090@mail.sustech.edu.cn), Liang Chen (liang.chen@chinafsl.com)

^*^ Author to whom correspondence should be addressed. Electronic mail: khli@sustech.edu.cn. Tel: (+86) 075588010176. FAX: (+86) 075588010197.

*^2^Foshan Electrical and Lighting Company Ltd., Foshan 528000, China.*

*^#^The authors contribute equally*


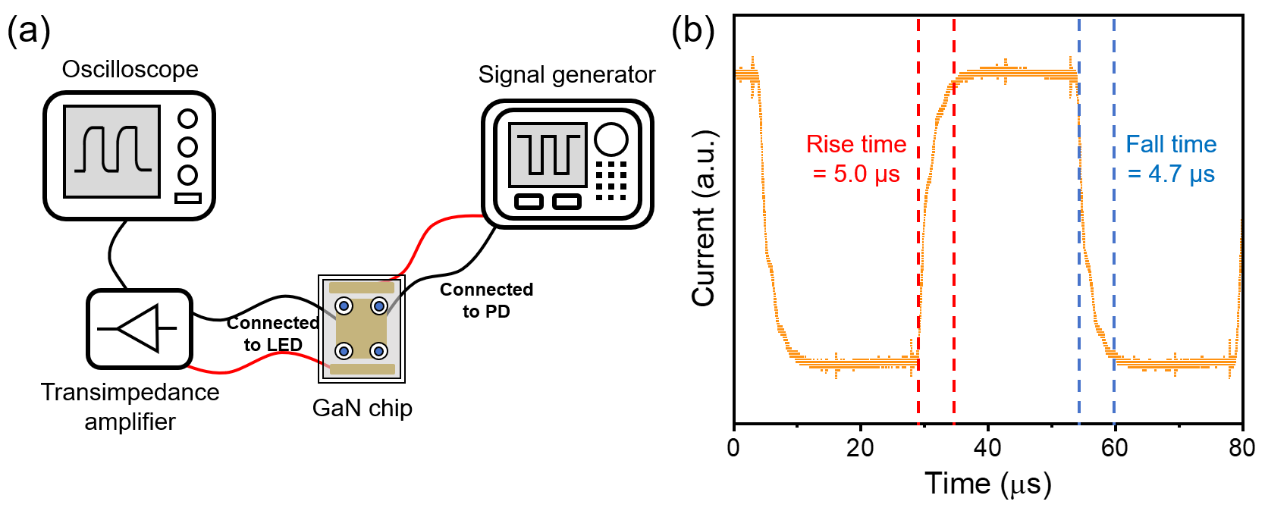


**Fig. S1** (a) Schematic diagram showing the experimental setup for measuring the transient response of the GaN chip. (b) Response of the GaN chip under a 20 kHz square signal.


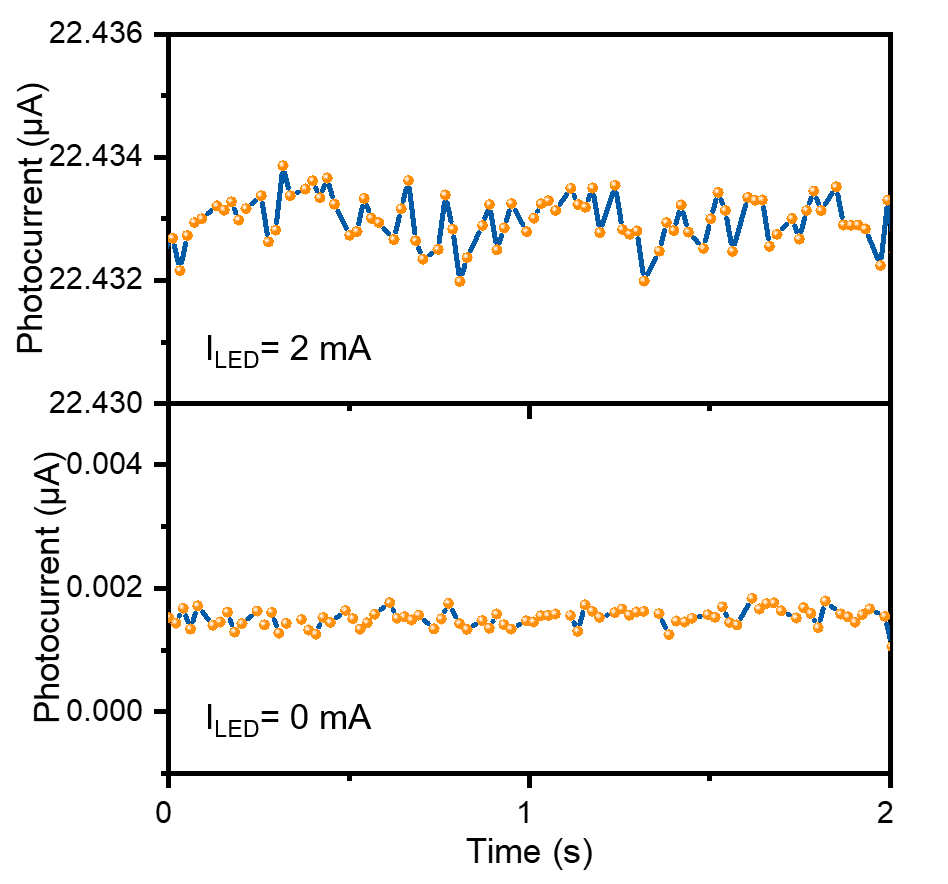


**Fig. S2** Plot of the photocurrent signal of the PD when the LED operates at 0 mA and 2 mA. The noise magnitude is found to be in the order of 10^-9^-10^-10^ A.


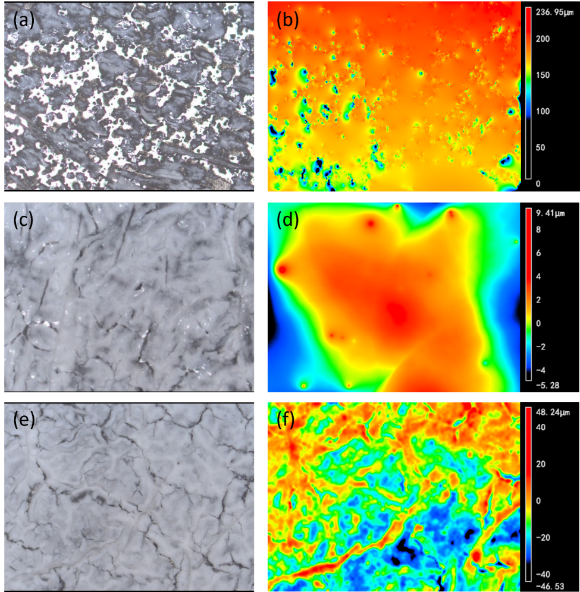


**Fig. S3** (a) Optical image and (b) 3D topography of FSP-PDMS film with a 5.8-μm-thick FSP coating captured by a confocal microscope. (c) Optical image and (d) 3D topography of FSP-PDMS film with a 10.1-μm-thick FSP coating captured by a confocal microscope. (e) Optical image and (f) 3D topography of FSP-PDMS film with a 14.8-μm-thick FSP coating captured by a confocal microscope.


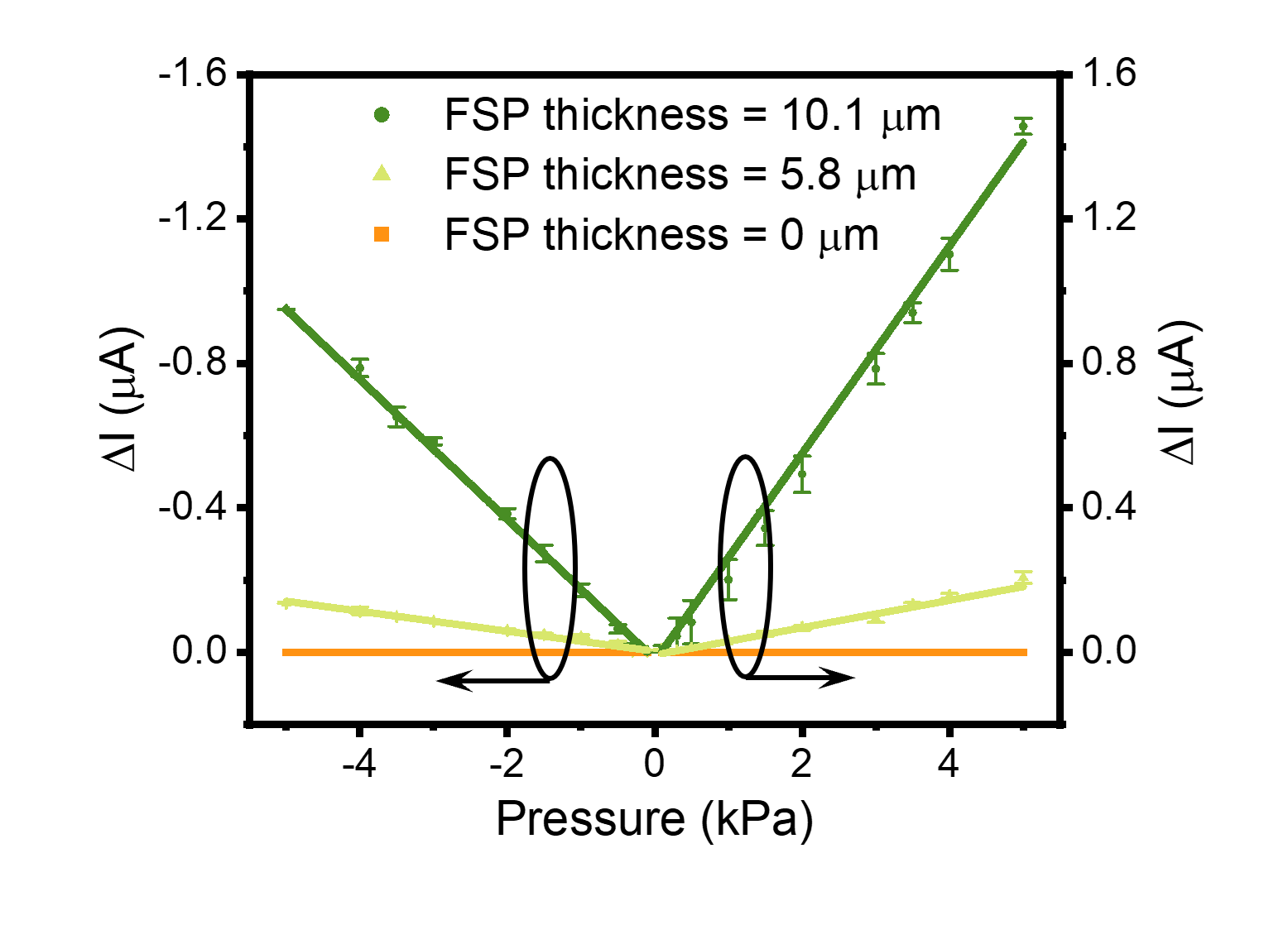


**Fig. S4** Comparison of effects of different thicknesses of the FSP coating on the device response.


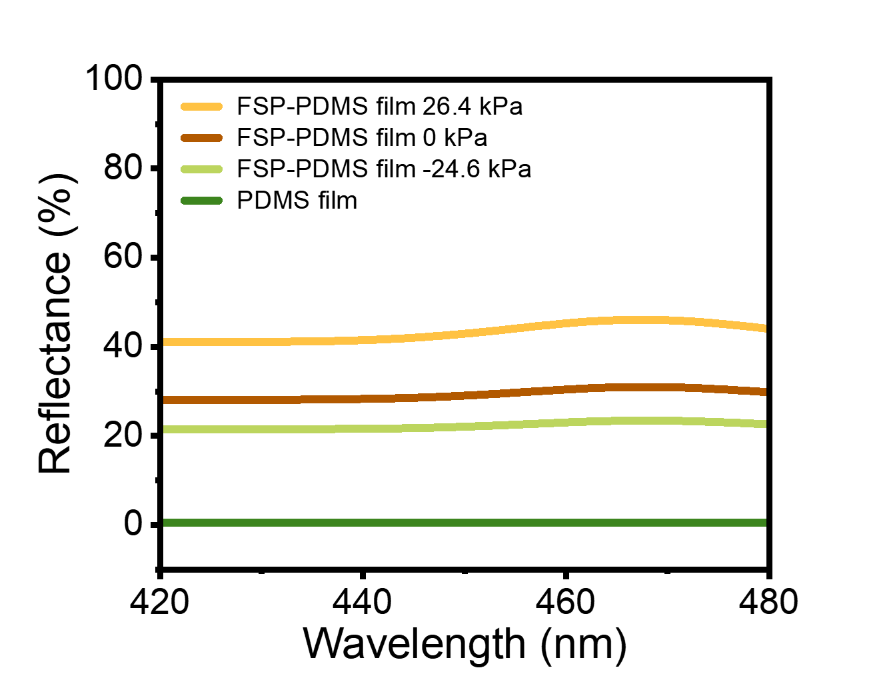


**Fig. S5** Reflectance spectra of the PDMS film and FSP-PDMS film under different air pressures.


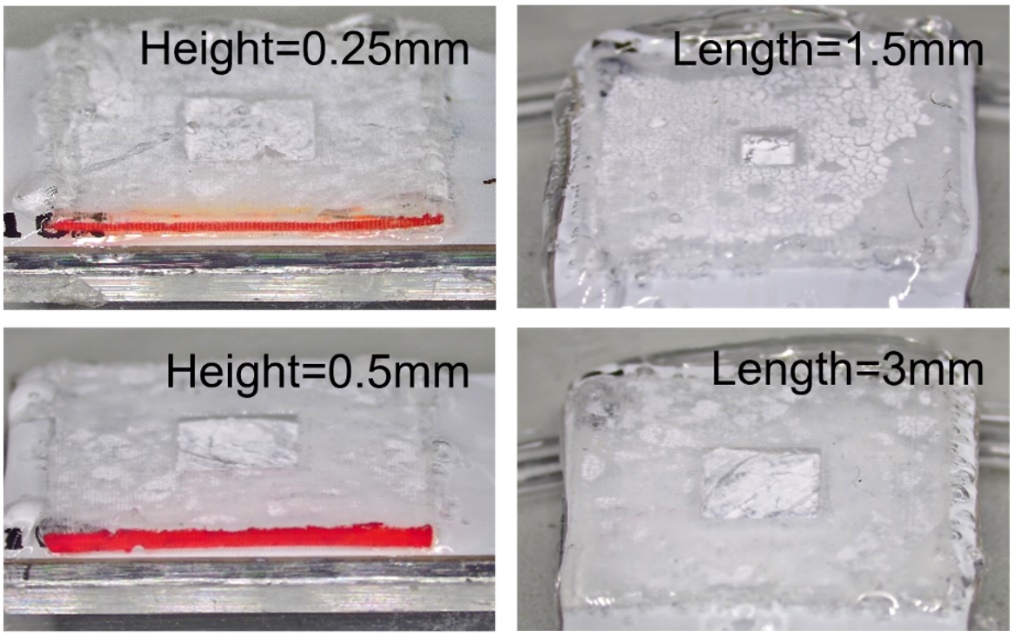


**Fig. S6** Optical images of the FSP-PDMS films with different combinations of heights and lengths.

**
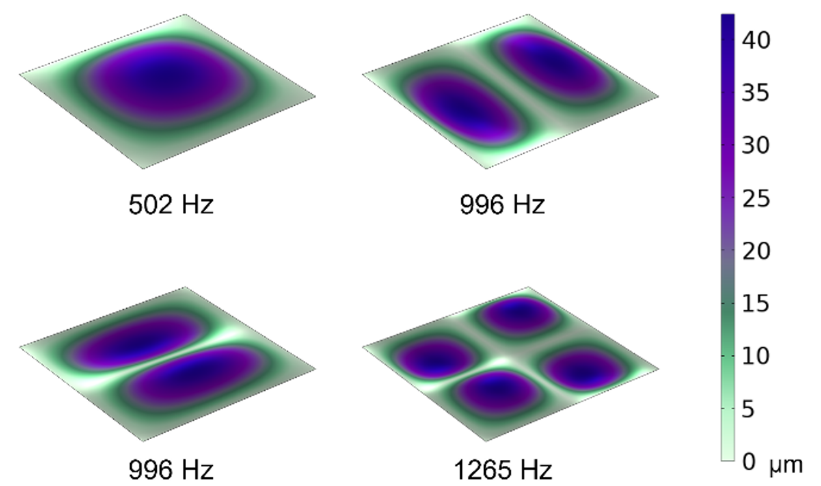
**

**Fig. S7** Simulation results showing the modal shape of the film at different resonant frequencies. The 3-D simulation is constructed based on the experimental film structure with the suspended region of 3 mm×3 mm and a thickness of 7 μm. Young’s modulus, density, and Poisson’s ratio are input to be 0.75×10^6^ Pa, 970 kg/m^3^, and 0.48, respectively^1–3^.

**
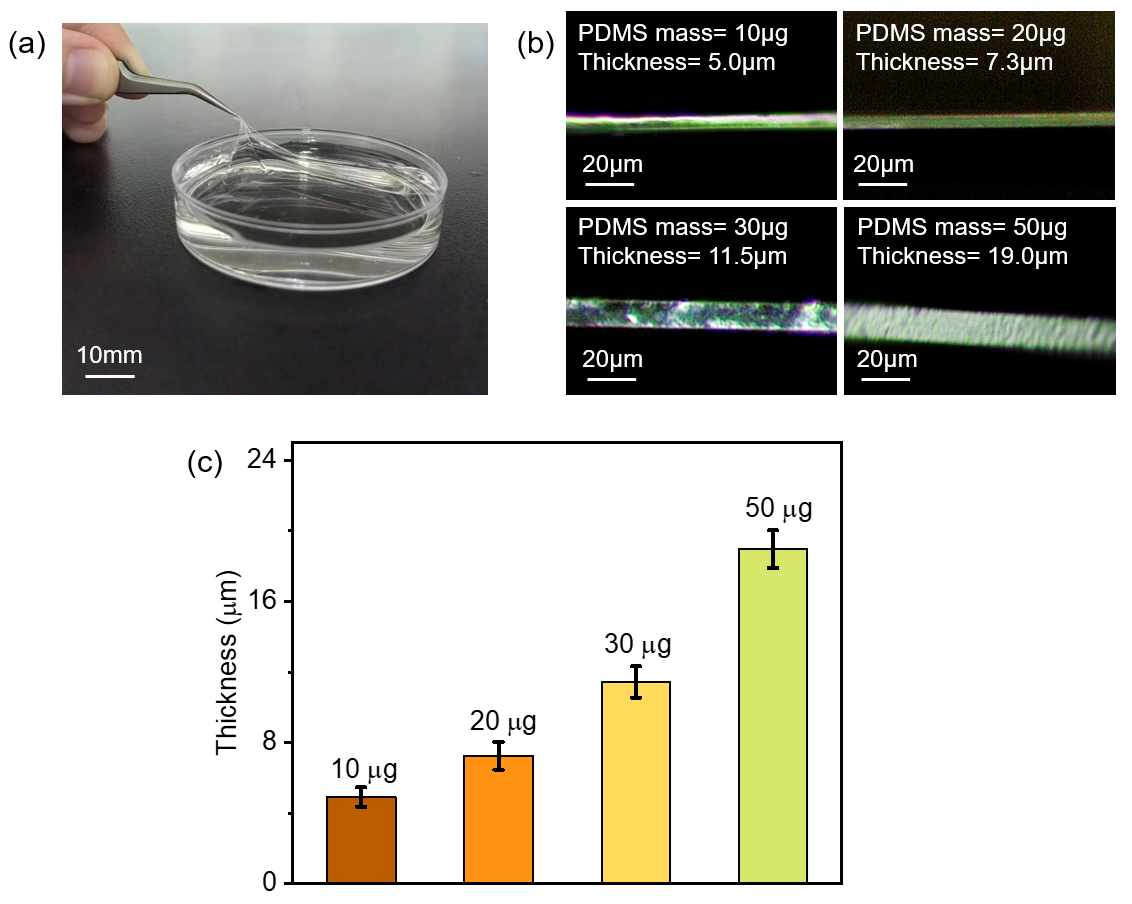
**

**Fig. S8** (a) Optical image of the PDMS film fabricated by the floating-on-water method. (b) Cross-sectional images of the PDMS films fabricated by applying different masses of PDMS. (c) Plot of the thickness of the PDMS film prepared by applying different masses of PDMS.

**References**

1. Babu, I. & De With, G. Highly flexible piezoelectric 0–3 PZT–PDMS composites with high filler content. *Compos. Sci. Technol.* **91**, 91–97 (2014).

2. Zhang, S., Ge, C. & Liu, R. Mechanical characterization of the stress-strain behavior of the polydimethylsiloxane (PDMS) substate of wearable strain sensors under uniaxial loading conditions. *Sens. Actuators Phys.* **341**, 113580 (2022).

3. Dinh, T.-H.-N., Martincic, E., Dufour-Gergam, E. & Joubert, P.-Y. Mechanical Characterization of PDMS Films for the Optimization of Polymer Based Flexible Capacitive Pressure Microsensors. *J. Sens.* **2017**, 1–9 (2017).
